# *Announcement*: Congenital Heart Defect Awareness Week — February 7–14, 2017

**DOI:** 10.15585/mmwr.mm6604a7

**Published:** 2017-02-03

Congenital Heart Defect Awareness Week is observed each year during February 7–14 to promote awareness and education about congenital heart defects (CHDs). CHDs affect approximately one in 100 births every year in the United States and are the most common type of birth defect (*1*,*2*). Heart defects are conditions that persons live with throughout their lives; an estimated 1 million children and 1.4 million adults in the United States were living with a CHD in 2010 (*3*). CDC’s website, Stories: Living with Heart Defects, includes personal stories by persons affected by CHDs (https://www.cdc.gov/ncbddd/birthdefects/stories/heartdefects.html).

CDC works to understand CHDs through initiatives that include working with state programs to improve newborn screening for critical CHDs, funding state programs to track birth defects, including CHDs, and launching projects focused on tracking children, adolescents, and adults with CHDs to make improvements in medical treatments and quality of life. CDC also provides funding for several research centers across the nation to help understand the causes of birth defects, including CHDs.

CDC-funded research recently reported associations for certain CHDs in infants of mothers who were exposed to pesticides at work (*4*) and a reduction in CHD risk for mothers with better diet quality (*5*). CDC research also found that children with CHDs receive special education more often than do children who do not have birth defects (*6*). CDC’s congenital heart defects website has additional information about CHDs (https://www.cdc.gov/ncbddd/heartdefects).
